# Intra-Parenchymal Renal Resistive Index Variation (IRRIV) Describes Renal Functional Reserve (RFR): Pilot Study in Healthy Volunteers

**DOI:** 10.3389/fphys.2016.00286

**Published:** 2016-07-06

**Authors:** Sara Samoni, Federico Nalesso, Mario Meola, Gianluca Villa, Massimo De Cal, Silvia De Rosa, Ilaria Petrucci, Alessandra Brendolan, Mitchell H. Rosner, Claudio Ronco

**Affiliations:** ^1^Institute of Life Sciences, Sant'Anna School of Advanced StudiesPisa, Italy; ^2^Department of Nephrology, Dialysis and Transplantation, San Bortolo Hospital and International Renal Research InstituteVicenza, Italy; ^3^Department of Clinical and Experimental Medicine, University of PisaPisa, Italy; ^4^Division of Nephrology, Center for Immunity, Inflammation and Regenerative Medicine, University of Virginia Health SystemCharlottesville, VA, USA

**Keywords:** renal functional reserve (RFR), renal resistive index (RRI), protein loading test, color doppler, intra-parenchymal renal resistive index variation (IRRIV), renal hemodynamics, renal blood flow, healthy volunteers

## Abstract

An increase of glomerular filtration rate after protein load represents renal functional reserve (RFR) and is due to afferent arteriolar vasodilation. Lack of RFR may be a risk factor for acute kidney injury (AKI), but is cumbersome to measure. We sought to develop a non-invasive, bedside method that would indirectly measure RFR. Mechanical abdominal pressure, through compression of renal vessels, decreases blood flow and activates the auto-regulatory mechanism which can be measured by a fall in renal resistive index (RRI). The study aims at elucidating the relationship between intra-parenchymal renal resistive index variation (IRRIV) during abdominal pressure and RFR. In healthy volunteers, pressure was applied by a weight on the abdomen (fluid-bag 10% of subject's body weight) while RFR was measured through a protein loading test. We recorded RRI in an interlobular artery after application of pressure using ultrasound. The maximum percentage reduction of RRI from baseline was compared in the same subject to RFR. We enrolled 14 male and 16 female subjects (mean age 38 ± 14 years). Mean creatinine clearance was 106.2 ± 16.4 ml/min/1.73 m^2^. RFR ranged between −1.9 and 59.7 with a mean value of 28.9 ± 13.1 ml/min/1.73 m^2^. Mean baseline RRI was 0.61 ± 0.05, compared to 0.49 ± 0.06 during abdominal pressure; IRRIV was 19.6 ± 6.7%, ranging between 3.1% and 29.2%. Pearson's coefficient between RFR and IRRIV was 74.16% (*p* < 0.001). Our data show the correlation between IRRIV and RFR. Our results can lead to the development of a “stress test” for a rapid screen of RFR to establish renal susceptibility to different exposures and the consequent risk for AKI.

## Introduction

The assessment of the risk for renal injury in a given patient is a key determinant in developing strategies for renal protection. Although glomerular filtration rate (GFR) is the parameter most used to assess renal function, it is not able to express the capacity of the kidney to increase filtration in response to specific stimuli. However, changes in urea excretion rate following different provocative stimuli were first demonstrated in 1923 (Addis and Drury, 1923) and since then, these changes in kidney function in response to various stimuli have proven useful in assessing renal reserve which may be a key determinant in the risk for kidney injury after a stress exposure (either hemodynamic or nephrotoxic) (Sharma et al., 2014; Husain-Syed et al., 2015).

At present, it is generally accepted that animal protein ingestion, which represents a kidney stress test, can induce a large rise in GFR likely through modifications in renal hemodynamics (Sharma et al., 2014, 2016). The degree of GFR variation in response to such measures represents the renal functional reserve (RFR). Studies have demonstrated that RFR, while still present, is progressively reduced in worsening stages of chronic kidney disease (CKD) (Bosch et al., 1984; Rodrìguez-Iturbe et al., 1985; Ter Wee et al., 1987) as well as in the elderly (Böhler et al., 1993). On the other hand, GFR may be maximized in certain hyperfiltration states such as diabetic nephropathy and thus cannot increase further in response to a protein load (Zeier et al., 1992).

The exact mechanism by which GFR increases in response to such stress stimuli is not completely understood and different hypotheses are still under investigation. For instance, there may exist a population of “dormant cortical nephrons” (not involved in filtration during resting conditions) potentially recruitable in response to a stress such as protein loading. Glomerular hyperfiltration is another potential mechanism. In most studies, the filtration fraction appears to be constant suggesting a change in blood flow as a primary mechanism (Hostetter, 1986; Sølling et al., 1986) while some authors found an increase of transcapillary hydraulic pressure gradient (Chan et al., 1988; Rodrìguez-Iturbe et al., 1988) that would result in increased GFR. While these mechanisms may be operative, the final common mechanism involved in RFR is likely afferent vasodilation (Hostetter, 1986; Sølling et al., 1986; Chan et al., 1988; Rodrìguez-Iturbe et al., 1988). This is supported by the temporal changes observed between alterations in renal hemodynamics after a protein load (within the 1 h) and the subsequent maximum rise in GFR (about 2–2.5 h) (Bosch et al., 1983; Sølling et al., 1986).

The intra-parenchymal renal resistive index variation (IRRIV) test relies on a mechanical abdominal stress, consisting of compressing renal arteries and veins through an externally applied pressure and consequently reducing blood flow, which secondarily activates the autoregulation mechanism. This leads to afferent vasodilation in order to maintain glomerular perfusion (Villa et al., 2016).

Afferent vasodilation is the shared element of the two aforementioned provocative maneuvers and can be assessed by color Doppler (CD) ultrasound in the case of the mechanical stress test (IRRIV). The renal resistive index (RRI), is probably the most commonly used method to evaluate blood flow in kidney vessels (Tublin et al., 2003). As RRI calculates the relationship between systole and diastole, it is an indicator of flow resistance within the kidney and thus a valuable tool in assessing changes in renal perfusion. In this setting, a drop of RRI in an interlobular artery in a single patient may be considered as an indirect measurement of pre-glomerular vasodilation. Therefore, in the present physiological study, we hypothesized that IRRIV could be related to the RFR and serve as an easy bedside predictor of RFR.

## Materials and methods

### Subjects

Adult healthy volunteers were considered eligible for the study. Inclusion criteria were: (i) age more than 18 years old, (ii) no known comorbidities, (iii) normal blood pressure values, according to practice guidelines for the management of arterial hypertension of European Society of Hypertension (ESH) and European Society of Cardiology (ESC) (Mancia et al., 2013), and (iv) baseline estimated GFR, calculated using the Modification of Diet in Renal Disease (MDRD) equation, higher than 80 ml/min/1.73 m^2^. Exclusion criteria were: (i) chronic therapy that may modify renal blood flow and/or GFR (Angiotensin Converting Enzyme-Inhibitors, Angiotensin Receptor Blockers, beta-blockers, calcium channel blockers, loop diuretics etc.) and/or (ii) nonsteroidal anti-inflammatory drugs (NSAIDs) in the 2 days before the tests, (iii) ultrasound evidence of morphological kidney abnormalities and/or renal artery stenosis.

This study was performed under the ethical principles of the Declaration of Helsinki. The protocol was approved by the local Institutional Review Board. All participants were informed of the objectives of the study.

### Baseline RRI measurement

The RRI measurement was performed by one trained sonographer using a multi-frequency convex probe (Toshiba's Xario™ 200) and with an appropriate machine setting (Doppler gate around 2–4 mm, the lowest pulse repetition frequency without aliasing, the highest gain without obscuring background noise and the lowest wall filter) (Tublin et al., 2003). Since difference between automatic and manual RRI measurements has been found (Unal et al., 2004), we decided to use manual RRI calculations for all cases, according to our routinely clinical practice and in order to avoid an increased error ratio to the results. The RRI were calculated with the following formula: RRI = [(peak systolic velocity–end diastolic velocity)/peak systolic velocity], in which peak systolic velocity and end diastolic velocity were measured in the same wave. The RRI were calculated on three interlobular arteries (superior, middle, and inferior) in each kidney. Then, the average value for each kidney was recorded. We decided to measure all RRI in inter-lobular instead of inter-lobar or segmental arteries in order to evaluate vasculature near to the glomerulus. Intra-observer reproducibility of measurements was assessed; the intra-class correlation coefficient was 0.95–0.97 with an expected variability of ≤ ±3%.

### Protein loading test

All subjects were on a standard diet with 0.9 g of protein/Kg of body weight/day. The last meal was taken 12 h before. RFR was measured by means of an oral protein loading test (1g of protein/Kg of body weight) performed with cooked beef. Urinary creatinine (uCr) and serum creatinine (sCr) were measured by enzymatic method (IL testTM Instrumentation®, Laboratory SpA, Milano, Italy) and by ILab650 (Instrumentation Laboratory, Werfen Group, Barcelona, Spain). Creatinine clearance (CrCl) was calculated and corrected for 1.73 m^2^ of body surface area (BSA) as follow:

CrCl = uCr (mg/dL)/sCr (mg/dL)^*^ urinary volume (mL)/time (minute)^*^ 1.73/BSA (m^2^) The mean value between two measurements of 1-h CrCl obtained in resting condition was considered the baseline CrCl. 1-h CrCl was assessed in the following 4 h after protein load. The difference between the higher CrCl obtained after protein load and the baseline CrCl defined RFR.

In addition, in a subgroup of eight subjects, RRI were measured each 5 min from 30 min to 1 h after protein load, in order to evaluate alterations in renal hemodynamics, as suggested by previous studies (Bosch et al., 1983; Sølling et al., 1986). The lowest RRI reached during this period was taken as reference (post-protein load RRI). The difference between baseline RRI and post-protein load RRI was calculated and expressed as percentage.

### IRRIV test

IRRIV test was performed in all subjects, in supine position and following a rest of at least 5 min. A saline bag was applied on abdominal wall.

A dose response test was performed because of it was the first time that IRRIV test was used. In a subgroup of five healthy volunteers, RRI were recorded during the application on the abdomen of progressively heavier saline bags (from 5 to 20% of subject's real body weight, gradually increasing by 2.5%). A generalized estimating equation (GEE) multiple regression analysis was performed taking into account the weight of subjects, the different abdominal weight used for the test and their interactions. The selected weight for IRRIV test was the lowest weight associated with the maximum RRI reduction; this occurred when the estimation of the regression slope, considering the current weight and the heavier, is closest to zero and the *p* > 0.05. The GEE multiple regression performed showed that a weight equal to 10% of subject's real body weight was the lowest that obtains an angular coefficient close to zero (−0.0002) and a *p* value equal to 0.26 (Supplementary Figure 1).

According to this result, the weight of the bag was calculated as 10% of subject's real body weight. We recorded RRI in a middle interlobular artery each minute for the 10 min of mechanical abdominal stress to assess the change in RRI related to the compression of renal arteries and veins and the consequent reduction of blood flow. The lowest RRI reached during mechanical abdominal stress was taken as reference (stress RRI). The IRRIV was defined as the percentage difference between baseline RRI and stress RRI.

The difference between two RRI values was considered significant only in the case in which it was higher than 0.05, according to CD indirect criteria of renal artery stenosis diagnosis utilized by some authors (Krumme et al., 1996).

### Safety analysis

The intrarenal blood flow has been continuously monitored during the mechanical abdominal stress test, in order to recognize the incidence of potentially harmful hypoperfusion conditions.

Furthermore, the occurrence of clinical and/or subclinical AKI has been evaluated through the measurement of sCr (according to Kidney Disease Improving Global Outcomes (KDIGO) criteria) (Kellum et al., 2012) and urinary Neutrophil Gelatinase-Associated Lipocalin (uNGAL) (Mishra et al., 2005). Urinary NGAL was determined by the ARCHITECT® urine NGAL assay (Abbott Laboratories–Abbott Park, IL, USA).

### Statistical analysis

A descriptive analysis of the sample of the study was performed using Stata12. The potential error for each RRI measurement, based on the expected variability of the operator, was firstly calculated. Normality of variable distribution was tested by Shapiro-Wilk *W*-test. The correlation of RFR and IRRIV was assessed using Pearson's correlation coefficient. We performed a linear regression analysis using the RFR as the response variable and the IRRIV as the exploratory variable. In the subgroup of eight subjects, the correlation between IRRIV and the maximum percentage RRI reduction after protein loading test was assessed using Pearson's correlation analysis and linear regression analysis. All the results were considered statistically significant if *p*-value was found to be less than 0.05.

## Results

Thirty healthy adult volunteers were enrolled for this study. The characteristics of subjects, at baseline and during stresses, are shown in Table 1.

**Table 1:** **Main characteristics of the population of the study**.

**Baseline data**	**Entire Cohort (*n* = 30)**
Sex (male)	14 (46.6)
Age (years)	38 ± 14
Height (cm)	170 ± 9
Weight (Kg)	65.5 ± 12.6
BMI (Kg/m^2^)	22.4 ± 2.9
BSA (m^2^)	1.76 ± 0.2
Baseline sCr (mg/dL)	0.85 ± 0.18
Baseline CrCl (mL/min/1.73 m^2^)	106.2 ± 16.4
Coronal diameter (mm)	109 ± 6
Cortical thickness (mm)	17 ± 2
RRI baseline	0.61 ± 0.05
**Results**	**Entire Cohort (*n* = 30)**
RFR (mL/min/1.73 m^2^)	28.9 ± 13.1
Stress RRI	0.49 ± 0.06
Baseline RRI-Stress RRI	0.12 ± 0.04
IRRIV	19.6 ± 6.7

*Data are expressed as means (SD) or number (percent). BMI, body mass index; BSA, body surface area; sCr, serum creatinine; CrCl, creatinine clearance; RRI, renal resistive index; RFR, renal functional reserve; stress RRI, the lowest RRI reached during IRRIV test; IRRIV, intra-parenchymal renal resistive index variation. It is the difference between baseline RRI and stress RRI, expressed as percentage*.

We enrolled 14 male and 16 female subjects with a mean age of 38 ± 14 years old. The mean baseline sCr was 0.85 ± 0.18 mg/dl, while baseline CrCl was 106.2 ± 16.4 ml/min/1.73 m^2^. The RFR ranged between −1.9 and 59.7 ml/min/1.73 m^2^ with a mean value of 28.9 ± 13.1 ml/min/1.73 m^2^.

**Figure 1:**
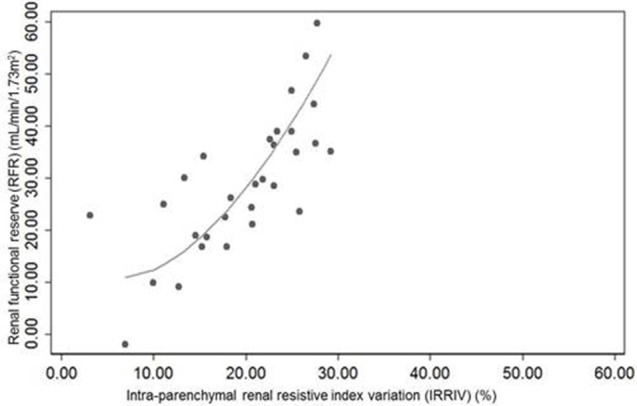
**Scatter plot of renal functional reserve and intra-parenchymal renal resistive index variation (IRRIV)**. IRRIV, is the difference between baseline renal resistive index (RRI) and stress RRI, expressed as percentage.

Morphometric and vascular characteristics of all kidneys ranged within normal values at ultrasound and CD examination. The mean baseline RRI was 0.61 ± 0.05, while the average value of mechanical stress RRI was 0.49 ± 0.06. The mean difference between baseline RRI and stress RRI was 0.12 ± 0.04, with a range between 0.04 and 0.19. Taking into account the maximum potential error in RRI measurement and based on the expected variability of the operator, 27/30 subjects had changes in RRI > 0.05. In one of them the test was considered negative and indicative of absence of RFR (Supplementary Table 1). In another subject the RFR was present but lower than the one measured in the others, while in the last case, it was not possible to identify particular characteristics to explain the result. The mean value of IRRIV was 19.6 ± 6.7%, with a range between 3.1 and 29.2%. Normality of variable distribution was tested by Shapiro-Wilk *W*-test. Pearson's correlation coefficient between RFR and IRRIV was 74.16% (R + 0.74, *p* < 0.001). The correlation between RFR and IRRIV was lower when RFR was less than 10 ml/min/1.73 m^2^, indicative of subjects without RFR, and greater than 50 ml/min/1.73 m^2^. While the ranges of RFR may vary widely, IRRIV may reach a plateau value. On the basis of the linear regression model performed, we found that an increase of IRRIV was correlated to an increase of RFR (coef 1.46, interc 0.28, *p* < 0.001, 95% CI: 0.95; 1.97, R + 0.74). The scatter plot of RFR and IRRIV is shown in Figure 1.

In the subgroup of subjects in which RRI were measured after protein load, Pearson's correlation coefficient between IRRIV and the maximum percentage RRI reduction after protein loading test was 0.76 (*p* = 0.03). The linear regression analysis performed showed that an increase of the maximum percentage RRI reduction after protein loading test was correlated to an increase of IRRIV (coef 0.87, interc 0.45, *p* = 0.03, 95% CI: 0.12; 1.63, R + 0.76).

Finally, the intrarenal blood flow analysis revealed the presence of a measurable pulsatile flow during the entire mechanical abdominal stress test, allowing us to calculate in each moment the RRI. Furthermore, the average value of sCr before and after the stress tests was 0.85 ± 0.18 and 1 ± 0.19 mg/dL, respectively, with a mean increase of 0.16 ± 0.07 mg/dL; the mean value of uNGAL at the baseline and after the stress tests was 4.7 ng/mL and 5.6 ng/mL, respectively.

## Discussion

Our results show that IRRIV during mechanical abdominal pressure correlates with RFR. We hypothesize that the pathophysiological bases of this correlation may represent important implications in the diagnosis of subclinical kidney dysfunction or underlying CKD. In fact, while CKD stages 3, 4, 5 can be easily diagnosed by a reduction in GFR, stages 1 and 2 often present a normal baseline GFR and the diagnosis may only be made by a “stress test” such as protein loading.

The basis of the diagnostic power of the stress test rests in the hypothesis that renal autoregulation and the rise of GFR after a protein load have, as common element, afferent arteriole vasodilation. In the present study, this assumption has been verified through the finding of a positive significant correlation between vasodilation after mechanical abdominal pressure and vasodilation after protein loading test. In hyperfiltration states as well as in states where significant fibrosis has occurred, this auto-regulatory capacity, similarly to RFR, may be lost. The intrinsic capability of the kidneys to maintain constant renal blood flow and GFR over a wide range of perfusion pressure is mainly due to a myogenic adaptive mechanism and tubule-glomerular feedback (Robertson et al., 1972; Navar et al., 1982). These two systems, globally known as renal autoregulation, are not mutually exclusive and they act together to achieve a combined stabilization of renal function during changes in blood pressure (Loutzenhiser et al., 2006; Just, 2007). Myogenic control of renal vascular resistance has been estimated to contribute up to 50% of the total auto-regulatory response (Just, 2007). It usually occurs very rapidly, reaching a full response in 3 to 10 s (Just et al., 2001); it involves all pre-glomerular resistance vessels (Walker et al., 2000) and is observed even when tubule-glomerular feedback is inhibited by furosemide (Just et al., 2001) or by resection of the papilla (Walker et al., 2000). The myogenic mechanism is altered in hyperfiltration states, e.g., diabetic nephropathy (Hayashi et al., 1992), solitary kidney after nephrectomy (Pelayo and Westcott, 1991), arterial hypertension (Hayashi et al., 1989, 1992) and high salt diet (Takenaka et al., 1992).

Because renal perfusion pressure derives from the difference between mean arterial pressure and venous pressure, and intra-abdominal pressure largely increases venous pressure, it is reasonable that intra-abdominal hypertension may trigger the auto-regulatory mechanism via a change in effective renal perfusion pressure. This is the rationale behind the IRRIV. Kidneys may be particularly prone to develop hypoperfusion in this situation, because of their retroperitoneal position (Mohmand and Goldfarb, 2011). In animal models (Harman et al., 1982; Barnes et al., 1985) and humans (Bradley and Bradley, 1947), intra-abdominal hypertension leads to a linear decrease of renal blood flow. When intra-abdominal hypertension and renal hypoperfusion are prolonged, the myogenic mechanism is exceeded by the activation of Renin-Angiotensin-Aldosterone System which ultimately leads to a contraction of arterioles and the subsequent increase of renal vascular resistance (Harman et al., 1982). The important link between intra-abdominal pressure and kidneys is also underlined by several clinical trials that show acute kidney injury (AKI) may result even from low degrees of intra-abdominal hypertension, often observed in critically ill patients (Dalfino et al., 2008; Vidal et al., 2008). Moreover, intra-abdominal hypertension and low abdominal perfusion pressure were demonstrated to significantly predict the development of AKI (Vidal et al., 2008). External mechanical abdominal pressure, through the compression of renal arteries and veins and the consequent decrease in blood flow, can activate autoregulation mechanism and lead to pre-glomerular vasodilation in order to maintain renal blood flow and GFR. Therefore, the expected response to mechanical abdominal pressure is a rapid drop of renal intra-parenchymal resistances, identified in Doppler ultrasound measures with an unchanged peak systolic velocity, an increased end diastolic velocity and a reduction of RRI (Supplementary Figure 2). We hypothesized that IRRIV may well describe the actual RFR of subjects and be an easier test to perform bedside measure of renal reserve. In our study, Pearson's coefficient identified a positive significant correlation between RFR and IRRIV (*p* < 0.001). The Figure 1 shows that an increase of IRRIV was associated to a parallel increase of RFR in a given subject. According to physiology, while RFR may widely vary, IRRIV should reach a plateau value, thus interpretation of the stress test should consider the linear part of the curve. These preliminary results seem to confirm our hypothesis.

Our pilot study has several limitations, such as the relative small sample size. However, our findings are consistent with a recent population based study which assesses the distribution of RRI values in healthy volunteers based on sex and age, thus determining RRI reference values (Ponte et al., 2014). Moreover, we calculated the potential error for each RRI measurement, based on the expected variability of the operator (Supplementary Table 1). Also in case of maximum expected error, the difference between baseline and stress RRI was higher than the threshold value of 0.05 in all except three cases, two of them where RFR was absent or low (Krumme et al., 1996).

Formal inulin clearance studies were not performed. Nevertheless, a previous study found that CrCl and inulin clearance measured after protein load did not differ significantly, showing that creatinine is a safe and reliable marker for measuring GFR (Ronco et al., 1988).

Analysis on oxidative stress potentially caused by the transient kidney hypoperfusion are not available, furthermore a direct measurement of intra-abdominal pressure during the stress test has not been performed in our cohort of healthy volunteers. However, our preliminary results may suggest that IRRIV is an indirect, safe and easy to measure correlate of RFR. In particular, although a kidney hypoperfusion state might be induced during the mechanical abdominal stress test (due to the slight increase of intra-abdominal pressure), the presence of a measurable pulsatile flow has been observed during the entire test. None of the enrolled subjects presented a clinical or subclinical AKI. Nevertheless, this issue should be taken into account mainly in frail patients in which even a limited hypoperfusion state might be harmful.

A growing body of evidence is today proposing an association between RFR and susceptibility to AKI (Sharma et al., 2014; Husain-Syed et al., 2015). In particular, RFR measurement may reveal subclinical loss of renal mass, early phases of CKD and high susceptibility to toxic exposures. In addition, these results should be confirmed by future clinical studies focused on assessment of susceptibility to AKI using new biomarkers and in patients with comorbid conditions such as hypertension and diabetes. Because protein load may be cumbersome, a mechanical abdominal stress test could be a useful, alternative and simple test for a rapid screen of RFR in all conditions requiring assessment of renal function. If our results will be confirmed by further studies with a larger sample size, the IRRIV test could be a complementary test in screening patients undergoing surgical or medical potentially invasive procedures or exposure to toxic drugs. In particular, the identification of patient's susceptibility in developing AKI before a planned physical or metabolic insult might help the physician to personalize patient's treatment and/or management, potentially reducing the risk of AKI development. Moreover, it might further improves knowledge on renal pathophysiology, mainly if applied to immunohistology and metabolomic analyses on translational animal models, instead of to be limited to serum and urine analyses on healthy volunteers or specific subgroups of patients.

## Author contributions

CR was the designer of the manuscript and the senior author of the paper; SS projected the mechanical abdominal stress test and made all subsequent drafts of the manuscript; FN projected the mechanical abdominal stress test and provided substantial contribution to the discussion of results; MM provided expertise in the field of ultrasonography, contributing to improve the mechanical abdominal stress test; GV analyzed data; MD was responsible for the collection of biochemical data provided in the study; SD was involved in writing and editing the manuscript, including figures; IP reviewed/edited the manuscript; AB reviewed/edited the manuscript; MR provided expertise in the field of nephrology and reviewed/edited the manuscript. All authors read and approved the final version of this manuscript.

### Conflict of interest statement

The authors declare that the research was conducted in the absence of any commercial or financial relationships that could be construed as a potential conflict of interest.
