# Supplementary material for: Intra-Parenchymal Renal Resistive Index Variation (IRRIV) Describes Renal Functional Reserve (RFR): Pilot Study in Healthy Volunteers
Source: Front Physiol. 2016 Jul 6;7:286. doi: 10.3389/fphys.2016.00286 (PMC4933701; doi:10.3389/fphys.2016.00286)
Supplement: Supplementary file 1 [file Table1.DOC]

Supplementary Material

**Intra-parenchymal Renal Resistive Index Variation (IRRIV) Describes Renal Functional Reserve (RFR): Study in Healthy Volunteers**

Sara Samoni*^1,2^, Federico Nalesso^2^, Mario Meola^1,3^, Gianluca Villa^2^, Massimo De Cal^2^, Silvia De Rosa^2^, Ilaria Petrucci^1,3^, Alessandra Brendolan^2^, Mitchell H. Rosner^4^, Claudio Ronco^2^.

^1^Institute of Life Sciences, Sant’Anna School of Advanced Studies, Pisa, Italy.

^2^Department of Nephrology, Dialysis and Transplantation, San Bortolo Hospital and International Renal Research Institute, Vicenza, Italy.

^3^Department of Clinical & Experimental Medicine, University of Pisa, Pisa, Italy.

^4^Division of Nephrology, Center for Immunity, Inflammation and Regenerative Medicine, University of Virginia Health System, Charlottesville, VA, USA.

# *Correspondence: Sara Samoni: [**sara.samoni@sssup.it**](mailto:sara.samoni@sssup.it)

# Supplementary Figures

**Supplementary Figure 1.** Graph showing the results of dose response test performed on five healthy volunteers. Each volunteer underwent to the measurement of renal resistive index (RRI) during the application on the abdomen of progressively heavier saline bag (from 5% to 20% of subject’s real body weight, gradually increasing by 2.5%). In the graph are reported all measurements obtained in all five volunteers at each weight. A decrease in RRI was observed during increasing saline bags weight from 5% to 10% of subject’s real body weight, gradually increasing by 2.5% (rhombus). An additional increase of fluid-bag weight did not lead to a further variation of RRI (circles).

**Supplementary Figure 2.** Physiological variations during mechanical abdominal pressure documented by color Doppler ultrasound. Coronal scans of right kidney obtained through a lumbar approach along the midaxillary and posterior axillary line with subject in supine position. The velocity/time curve recorded in a middle interlobular artery under normal conditions showed a renal resistive index (RRI) equal to 0.57 (**A**). During the mechanical abdominal pressure, the RRI decreased to 0.46 (**B**).

# Supplementary Table

**Supplementary table 1.** Potential errors calculated for each measurement, based on the expected variability of the operator.

|  |  |  |  | 3%-range of baseline RRI | | 3%-range of stress RRI | | min baseline RRI –  max stress RRI |
| --- | --- | --- | --- | --- | --- | --- | --- | --- |
| N | baseline  RRI | stress  RRI | baseline RRI –  stress RRI | min  baseline RRI | max  baseline RRI | min  stress RRI | max  stress RRI |
| 1 | 0.50 | 0.45 | 0.05 | 0.493 | 0.508 | 0.443 | 0.457 | 0.036 |
| 2 | 0.65 | 0.50 | 0.15 | 0.640 | 0.660 | 0.493 | 0.508 | 0.133 |
| 3 | 0.67 | 0.55 | 0.12 | 0.660 | 0.680 | 0.542 | 0.558 | 0.102 |
| 4 | 0.58 | 0.46 | 0.12 | 0.571 | 0.589 | 0.453 | 0.467 | 0.104 |
| 5 | 0.63 | 0.55 | 0.08 | 0.621 | 0.639 | 0.542 | 0.558 | 0.062 |
| 6 | 0.65 | 0.50 | 0.15 | 0.640 | 0.660 | 0.493 | 0.508 | 0.133 |
| 7 | 0.57 | 0.48 | 0.09 | 0.561 | 0.579 | 0.473 | 0.487 | 0.074 |
| 8 | 0.65 | 0.46 | 0.19 | 0.640 | 0.660 | 0.453 | 0.467 | 0.173 |
| 9 | 0.57 | 0.45 | 0.12 | 0.561 | 0.579 | 0.443 | 0.457 | 0.105 |
| 10 | 0.55 | 0.47 | 0.08 | 0.542 | 0.558 | 0.463 | 0.477 | 0.065 |
| 11 | 0.62 | 0.45 | 0.17 | 0.611 | 0.629 | 0.443 | 0.457 | 0.154 |
| 12 | 0.54 | 0.39 | 0.15 | 0.532 | 0.548 | 0.384 | 0.396 | 0.136 |
| 13 | 0.58 | 0.42 | 0.16 | 0.571 | 0.589 | 0.414 | 0.426 | 0.145 |
| 14 | 0.60 | 0.45 | 0.15 | 0.591 | 0.609 | 0.443 | 0.457 | 0.134 |
| 15 | 0.62 | 0.51 | 0.11 | 0.611 | 0.629 | 0.502 | 0.518 | 0.093 |
| 16 | 0.64 | 0.47 | 0.17 | 0.630 | 0.650 | 0.463 | 0.477 | 0.153 |
| 17 | 0.64 | 0.49 | 0.15 | 0.630 | 0.650 | 0.483 | 0.497 | 0.133 |
| 18 | 0.59 | 0.50 | 0.09 | 0.581 | 0.599 | 0.493 | 0.508 | 0.074 |
| 19 | 0.75 | 0.65 | 0.10 | 0.739 | 0.761 | 0.640 | 0.660 | 0.079 |
| 20 | 0.60 | 0.49 | 0.11 | 0.591 | 0.609 | 0.483 | 0.497 | 0.094 |
| 21 | 0.62 | 0.48 | 0.14 | 0.611 | 0.629 | 0.473 | 0.487 | 0.124 |
| 22 | 0.64 | 0.50 | 0.14 | 0.630 | 0.650 | 0.493 | 0.508 | 0.123 |
| 23 | 0.58 | 0.54 | 0.04 | 0.571 | 0.589 | 0.532 | 0.548 | 0.023 |
| 24 | 0.63 | 0.50 | 0.13 | 0.621 | 0.639 | 0.493 | 0.508 | 0.113 |
| 25 | 0.63 | 0.56 | 0.07 | 0.621 | 0.639 | 0.552 | 0.568 | 0.052 |
| 26 | 0.56 | 0.42 | 0.14 | 0.552 | 0.568 | 0.414 | 0.426 | 0.125 |
| 27 | 0.51 | 0.38 | 0.13 | 0.502 | 0.518 | 0.374 | 0.386 | 0.117 |
| 28 | 0.58 | 0.43 | 0.15 | 0.571 | 0.589 | 0.424 | 0.436 | 0.135 |
| 29 | 0.65 | 0.63 | 0.02 | 0.640 | 0.660 | 0.621 | 0.639 | 0.001 |
| 30 | 0.65 | 0.55 | 0.10 | 0.640 | 0.660 | 0.542 | 0.558 | 0.082 |

Baseline and stress renal resistive index (RRI) are presented for each subject. Considering the maximum potential error deriving from operator variability, ranges of baseline and stress RRI are expressed as minimum and maximum values. In particular, minimum and maximum potential errors were calculated as baseline RRI ± baseline RRI*0.03/2 and stress RRI ± stress RRI*0.03/2. The min baseline RRI – max stress RRI was calculated as the difference between the minimum baseline and the maximum stress RRI potentially obtainable. All min baseline RRI – max stress RRI, except only three cases, result higher than 0.05, assessing the robustness of the results.

RRI, renal resistive index.
