# Supplementary figures and images for: Intra-Parenchymal Renal Resistive Index Variation (IRRIV) Describes Renal Functional Reserve (RFR): Pilot Study in Healthy Volunteers
Source: Front Physiol. 2016 Jul 6;7:286. doi: 10.3389/fphys.2016.00286 (PMC4933701; doi:10.3389/fphys.2016.00286)

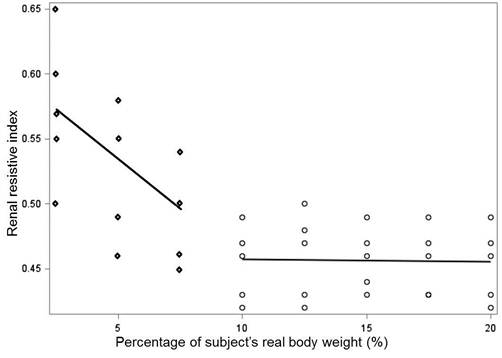

Supplement: Supplementary file 2 [file Image1.jpg]

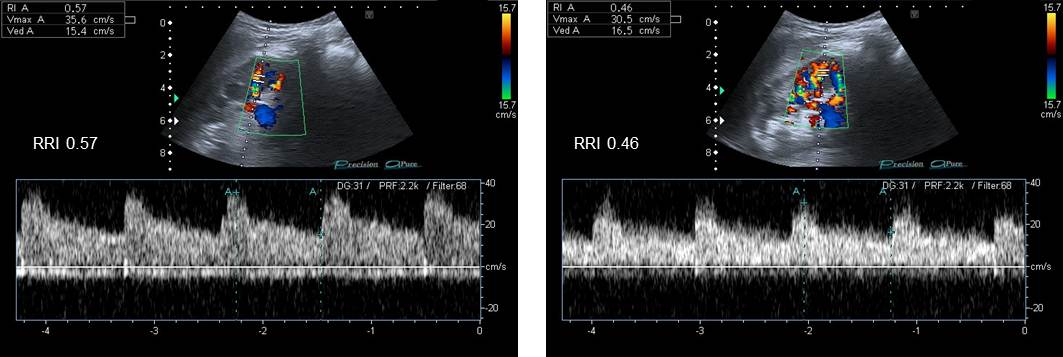

Supplement: Supplementary file 3 [file Image2.jpg]
